# Sport4Me: A people focused approach to engaging Australians in sport

**DOI:** 10.3389/fspor.2022.1087182

**Published:** 2023-01-06

**Authors:** Rochelle Eime, Hans Westerbeek, Shane Pill, Lindsey Reece

**Affiliations:** ^1^ Institute for Health and Sport, Victoria University, Victoria, VIC, Australia; ^2^ Physical Activity and Sport Insights, Research and Innovation, Federation University, Victoria, VIC, Australia; ^3^ College of Education, Psychology and Social Work, Flinders University, Bedford Park, South Australia, Australia; ^4^ SPRINTER, Prevention Research Collaboration, Sydney School of Public Health, The University of Sydney, Sydney, NSW, Australia

**Keywords:** sport, participation, community, retention, reengage

## Abstract

The traditional model of community club-based sport is fine for those, particularly children and youth, who enjoy the competitive focus and have the skills and commitment to play. But societal preferences during leisure time have changed dramatically over recent decades. However, sport organisations have made limited progress in response to these changes in providing ways in which children and youth can participate outside the traditional competitive structures and environments. In this paper the context of community club-based structures is reviewed leading into an assessment of the associated impact of these structures on sport participation. Children and youth's current motivations to play sport including what makes sport fun to play, are considered. It is then demonstrated that the associations between motivations to play sport and the factors that contribute to fun and enjoyment, are often misaligned for many individuals, with a primary focus on competition-based structures to deliver community club sport. In the final part of the paper a model for community sport organisations where people are put first is proposed - Sport4Me. Sport4Me is about flexible, inclusive, equitable sporting opportunities that focus on friends, fun, physical literacy and play. The model would complement the traditional competitive club-based model and afford participants more choice whilst fostering an environment that promotes lifelong involvement in sport. This model will require structural and cultural changes to the sporting environment and include coaching practices. Sport4Me is an evidence-based model, but it is not radical in its conceptualisation but rather, builds on previously proposed approaches, considers the needs and wants of potential sport participants and widens the scope of sport delivery.

## Introduction

### The origins of club-based sport

Community participation in sport in many countries including Australia, was established through local sports clubs providing competitive formats. With the purpose of competitive sport in mind, clubs developed structures that would seek sporting talent for elite teams and as such developed pathways for children and young people to develop and master the skills of the game. In this paper we focus on the Australian sporting context, but it is important to review how community club sport came into being around the world to better understand its systemic roots.

Competitive club-based sport remains popular for children, however, significant drop-out during adolescence occurs, and few adults play (1). Half of all sport participants in community clubs are aged between 5–14 years (2). Most people eventually stop playing competitive sport, with around 45% dropping out within three years and mainly during adolescence (1). The Sport Participation Pathway Model (1) demonstrates the patterns of participation in organised, elite, non-organised sport and organised social and recreational sport-related activities across age groupings and displays the issue of dropout in competitive club-based sport, specifically for adolescents. Further, the SPPM demonstrates that organised social and recreational sport offerings are largely an untapped market with few options to play in this context (1).

Not all sport is consumed through sport clubs (3). Whilst children's participation in sport is largely through a local sport club or association, adults who play sport are much less likely to do so within a club-based setting because more flexible and less competitive options seem to be preferred by many adults (3). Also, organised sport is increasingly being offered by various private and public organisations that are not sport clubs (4).

### The Rijnland and Anglo-Saxon model of sport participation

The context of participation in sport around the world is largely along two dominant dimensions – community participation and elite participation. It has been recently reported that the sport system in Europe essentially evolved on a platform of amateur participation and recreation-based sport (5). De Jonghe (2019), in that regard, has argued that where the Anglo-Saxon model underpinning the American sporting system is dominated by private sport business operators concentrating on short-term commercial gain, the European-based Rijnland model focuses on amateur athletes playing the physical sport game and playing this within their communities (6). The emphasis is on play rather than prowess and fitness, and on community rather than media. In the Australian context, in that regard, engagement in sport has been and continues to be a cultural activity forming part of the imagined Australian identity. That is, sport was part of building Federation - a national identity. Sport, was an important way of establishing a sense of Anglo-Australian parity and part of Australia's colonial nationalism. Like in the Rijnland perspective, sport in Australia has historically also been imbued with a social purpose, of achieving a sense of community through a common vision of identity.

Andrews and Silk (2018) explain the Anglo-Saxon model of sport delivery from the perspective of underlying neoliberal values. They note that neoliberalism as a structure of feeling has influenced the development of “corporate sport” – which is the commercialised and spectacularised domain of elite and professional sport (p. 512) (7). They further describe corporate sport as an expression of contemporary late capitalism, to the extent that in this day and age, “virtually all aspects of the global sport institutions (governing bodies, leagues, teams, events, and individual athletes) are now un-selfconsciously driven and defined by the inter-related processes of: corporatization (the management and marketing of sporting entities according to profit motives); spectacularization [the primacy of producing of entertainment-driven (mediated) experiences]; and, commodification (the generation of multiple sport-related revenue streams) (p. 140) (8).

As such in the Anglo-Saxon model, the commercial delivery mode of sport dominates and with this, a focus on developing elite (corporate) sport. In more recent times these quite extreme models started to mix and merge, and in Australia, which can be classified as an advanced sport economy, a refined mix of these two approaches to producing sport has evolved. It also needs to be noted that with the ever-increasing commercialisation of sport globally, the influence of the Anglo-Saxon “capitalist” model of sport is becoming significant. With Australia's “hybrid” model, it may well be that such increasing commercialisation is also impacting the ways in which club-based sport in Australia is perceived to be delivered.

Community sport participation provided the foundation for well-established rules, regulations and traditions of a sport with the cycle of participation continuing from junior and youth participation to senior competitions and ultimately to elite and professional sport (9). In Australia, community club structures remain the foundation of Rijnland inspired sport systems and the backbone of Anglo-Saxon focused player pathways into professionalised talent identification and elite competitions (9, 10).

One of the defining features of sport and elite sport in particular, is the desire to “win”, and this often leads to bias towards finding the highest skilled players (11), and to increasing competitiveness (12). In terms of inclusion of all of those who want to participate, the problem is often that (club) sport organisations continue to focus on delivering competition formats and striving to be on top of the competition ladder, and an emphasis on winning (11). However, for many individuals, winning is not a major motivation to play sport, and competition-focused clubs may actually contribute to the decline in participation (11).

There are many sport models that have a focus on competition and elite development pathways and these have been recently explored and critiqued as they are not focused on community level sport (1). Further other models of sport such as sport for development (and peace) focuses on using sport and physical activity as a tool or social intervention to address broader issues such as empowerment, education, and employment (13). Other forms of sport such as informal sport have had limited progress due to sporting environmental culture, governance and practices (14). In these cases informal sports groups have had difficulty accessing sports facilities and there was tension between these informal groups and the local sports-clubs who were currently utilising the facilities for traditional training and competitions (14).

The traditional Rijnland participation values of sport, the ways in which sport is delivered and in which formats, are challenged by changing lifestyles (9). Sport organisations must respond to the changing landscape of participation (14). Understandably, to change club cultures from competition and winning focused cultures to more inclusive and broader participation based, is not an easy process.

An example of such slow change is the move towards more gender inclusive sporting environments. In the last decade sport organisations in Australia, mainly driven by government incentives and start-up professional sport competitions for females, have started to open up clubs and competitions for females (15, 16). However, most sport clubs in regard to their leadership and structures continue to focus on organising competitions which inhibits the inclusion of new participants and especially those with limited experience playing sport (11). This unfortunately is not a new phenomenon. In this paper we present supporting data and propose a new model of community sport delivery which is more inclusive and matches societies changing leisure-time priorities.

### Revisiting enduring issues – quality coaching and age-specific modified sport

Two decades ago, a “Children in Sport Committee” was established by the Australian Sports Commission. The Committee undertook 18 months of intensive consultation with state and territory governments, teachers, sports coaches, the Australian Council of Health Physical Education and Recreation (ACHPER), and numerous National Sporting Organisations. The Committee identified the six issues of widespread concern:

• low participation rates in sport activities;
• poor levels of skill development;
• a limited range of available sports;
• an adult orientation in many children's sports;
• limited opportunities for girls to fully participate in many sports; and,
• a lack of quality sport coaches. (17, 18).

The Aussie Sport program (1985–1995) featured modified sports to encourage national sport organisations to develop age-appropriate sport forms for more accessible sport (19). At this time, the National Aussie Sport Unit and the Australian Coaching Council identified that a common coaching approach, often described in literature as directive (20) “command and practice” style coaching (21) was contributing to the concerns identified by the Children in Sport Committee. This resulted in the Australian Sports Commission developing a new game-based and player-centred approach to coaching, called Game Sense (22). Resources developed by the Australian Coaching Council included coaching video (23), a coaching guide (24), Game Sense game cards (25), and many articles on the Game Sense approach in Australian Sports Commission publications such Aussie Sport Action and the Sport Coach journal (21). The Game Sense continues to underpin the Playing for Life Philosophy of Australian sport, and yet Game Sense, and contemporary coaching approaches generally, remain new or unknown to most community sport coaches (26).

With this common coaching approach enduring in community sport, concerns about the quality of coaching persist (27, 28), and participation is still in decline in club-based competitive sport when (potential) participants opt for a move towards more flexible and individual types of physical activity both pre-COVID (10) and exacerbated through COVID-19 (4). In Australia, the concept of modified sports to encourage national sport organisations to develop more accessible sport forms is not new, but a further focus on age-appropriate sport forms to meet leisure, recreation as well as competitive sport interests at any age is required.

### The broader needs of participants - key influencing factors

In order to drive participation in sport, we need to understand the key influencing factors. Many studies on determinants of physical activity and participation in sport have utilised a socio-ecological approach which looks at determinants across various domains including the intrapersonal, interpersonal, and organisational domains (29–33). These studies highlight that participation and retention in sport and physical activity is not related to a single factor such as competency but a range of factors across the different domains.

Parents, in that regard, are often a driving force when it comes to children deciding in which sport to partake (34). For most children, “sportlike activities” are a means to play and controlled by them, while club- based organised sport is something most adults want their children to do and is controlled by adults (35, 36). When children start playing their focus is on fun, yet the system is built for competition. Research suggests that early sport sampling in childhood is associated with a higher likelihood of recreational participation in adolescence, while early sport specialisation is associated with a higher likelihood of performance participation. Non-participation in sport in childhood is associated with an increased likelihood of non-participation in adolescence (37). To build a more physically active community, sport participation in childhood is a good starting point (10). School-age is generally a good age to start to play community club-based sport, if children are younger they generally are not developmentally ready and have higher rates of dropout (1).

When moving into playing sport as an adolescent, perceived and actual belief in competence and ability to play the sport begins to influence participation motivation. Specifically, sport competence may underlie general self-esteem, in that a sense of self-worth or self-esteem is determined by specific competencies such as sport ability (38). Entering adolescence, low movement ability makes it more likely that individuals become sedentary which in turn means a higher likelihood of becoming overweight (27, 39). Movement ability seems a key factor in keeping young people involved in physical activity. Additionally, entering adolescence, young people become increasingly sensitive to equal treatment and perceived fairness. What this means in the context of sport, is that the relationship young people have with the coach becomes a key determinant in participation (27).

Very few adults participate in club-based competitive sport (2). Participation decreases across the lifespan and for adults from 15% for those aged 20–24, to 7% (30–34 years), 6% (40–44 years), 4% (50–54 years), 2% (60–64 years) and 1% (70–74 years) (2). The patterns of participation in sport and in leisure-time physical activity differ considerably across the lifespan in both types of activities and settings for activity (3). For example, with regards to participation in sport, children are more likely to participate within a sports club or association setting compared to adults who were more likely to participate across many different settings including gyms, fitness centres and other community and work settings. Further, motivations to be active and play sport are further influenced by societal changes and the policy context in which sport is produced, delivered and consumed (10).

However, there remains an important place for competitive club-based sport. As such, in Australia a need to reconsider the pedagogy of sport coaching for engagement, enjoyment and performance has been recognised by national peak bodies for sport for some time (21, 22, 40). However, all levels of government, government agencies and sport organisations need to understand that the way in which many people want to consume and play sport is more diverse than being channelled into organised competition structures. Changing patterns of participation have been developing over the past couple of decades, and perhaps COVID-19 has been the “perfect storm” for stakeholder organisations to reassess, develop and implement a new model of sport (4). In order to consider what a new model of sport would look like, we first need to summarise what are the most important motivators for people to participate in sport.

### Motivations to play competitive club-based sport

As participation in sport differs considerably across the lifespan, so do the motivations to play sport (41, 42). Children and youth are more motivated to perform and compete and become a professional athlete, learn new skills and work towards a sense of achievement whereas adults are much less motivated by performance and competition and more by improving health and losing weight, and by being a good role model (41, 43, 44). Older adults are more motivated to play for social reasons and to be with their friends (41). The reality is that most individuals play sport for fun and engagement, for health and for social reasons, particularly adults and older adults (41, 43, 44).

Motivations to be physically active are important to understand and vary according to different types of activities and settings such as team-based activities compared to individual types of activities (45). Overwhelmingly, “to have fun” is the reason why people play sport across gender, age, region and playing characteristics (46–50). Fun can be described as intrinsic motivation to play sport (51). This is followed by physical health or fitness, performance and competition as well as for social reasons, to be with friends and a to generate sense of achievement (43, 44, 46).

Some gender differences include that males, more than females, are motivated to play sport to perform, compete and to be a professional athlete (46, 52–54). While most people, irrespective of gender, are motivated by intrinsic factors, it has been suggested that boys are more likely to be motivated by extrinsic factors in their choice to play sport such as a desire for recognition (55). Further, intrinsic motivations like enjoyment and socialising may enhance wellbeing, whereas a focus on extrinsic motivations like winning can negatively impact individual wellbeing (56).

Females are more motivated by intrinsic factors, such as playing sport to improve physical and mental health, to lose weight, to be a good role model, and to learn a new skill (41). For girls, the importance of the group context relative to individual physical self-concept and motivation appears important (57).

### Exploring fun and creating a memorable experience as key motivations to play sport

Since fun and enjoyment are often reported by participants irrespective of age as the main motivations to play sport across age and gender, we explore the meaning of fun and enjoyment further. Fun and enjoyment in that regard are also very important reasons to keep playing (retention) (10, 58). Eime et al., (2022) investigated what it meant “to have fun playing sport” and found that across all ages the main factors contributing to “fun” playing sport were “being challenged to improve” and “get better at sport” followed by “playing with friends” and “socialising” (46, 48, 49). These factors relate to skill development and social engagement (46).. Winning was less important as a fun factor with fewer than half of respondents indicating that “winning” was important for them to have fun (46).

For adolescents, the main factors contributing to having fun playing sport relate to “personal development” (such as trying your best), “social development” (playing well as a team) and “organisational factors” including “getting game time” and “well-organised practices” (46). For adults the main factor that makes playing sport fun was “keeping fit” (46).

Men and boys were more likely to have fun when “winning” compared to women and girls who were more likely to report have fun playing when they had a “friendly coach”. Further, adolescent girls reported “trying your best” and “parents behaving well”, where boys reported “getting playing time” and “well-organised practices” (46) as reasons to have fun. In summary, for women and girls the main “fun” factors related to social elements (coach and parents) whereas for boys and men the main factors related to game and practice elements (46).

Younger players were more likely to report having fun when they were challenged, when their coach was friendly and when they were winning whereas older players had more fun when socialising and playing with friends as well as when they were keeping fit (46).

Sport professionals, such as coaches, sport and recreational providers, and sport researchers, therefore, need to be careful when reciting “fun” as the motivator of sport participation because as explained, “fun” has various constituting components. Having “fun” is one of the main drivers that motivates people to play sport, but what “is” fun for one cohort is unlikely to be “fun” for another.

Kahneman (2010) suggested that we choose to do things based on the memory of the experience and the anticipation of the new memory that will be created (59). If that is the case, then what is important in influencing more people to play sport is the story that is created from the memory of the experience. Most of our experiences do not leave identifiable memories, they are lost or ignored. The question for sport coaches and sport providers in that regard, is, how do we create experiences that encourage the desirable anticipation of the new memory to be created. The significance of Kahneman's idea that our remembering self can feel very different about an event than what we feel at the time of experiencing it. That is, something may have moments of “fun”, but the memory of the experience may not be one of satisfaction with the experience or the outcome of the experience.

We suggest, people drop out of sport because of the absence of agency and choice leading to a lack of satisfaction with the experience and a loss or absence of anticipation of a new memory being created, the lack of satisfaction, loss or absence of an anticipated memory creates a memory attached to the emotions associated with this experience that may lead to choosing to do something other than continue with sport. Sharot (2017) explained that emotion persuades behaviour, and in so doing, determines the moments that become remembered (60). Creating a fun experience might therefore be about creating the anticipation of “good” memories, which is aided by persuading an emotional response associated with the needs satisfaction of the individual. Therefore a “one size fits all” community sport participation model will only offer the anticipation of a “competition and winning” memory of participation. This will leave only those interested in competition and winning driven sport structures with a high level of needs satisfaction.

### Sport4Me: A contemporary model for sport participation

**Figure 1:**
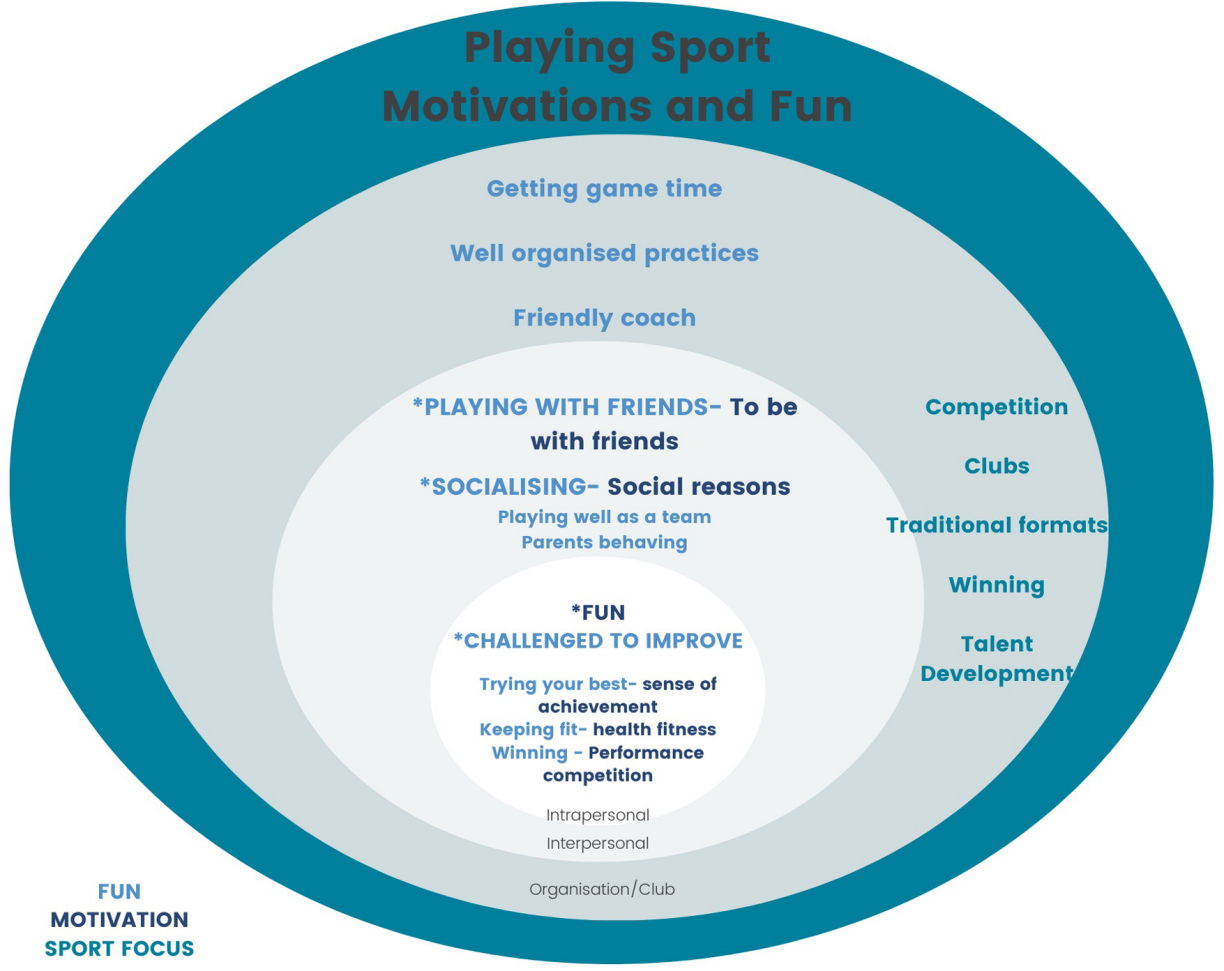
Sport structure, motivations and what makes playing fun.

In Figure 1, we use a socio-ecological approach to picture the Australian sporting system at the intrapersonal, interpersonal and organisational level (61, 62). In this figure we highlight the main motivations, and factors contributing to what “fun” means when playing sport. Future research can assist the further development and confirmation of these elements across different demographic groups.

For example, at the intrapersonal level, it is visualised that “fun” is at the heart of what motivates individuals to play sport, and that the main factor contributing to “fun” is “being challenged to improve”. It is shown that “having fun playing sport” is derived from having a sense of achievement and trying your best, and that the health and fitness motive is linked to fun by “keeping fit”. Finally, performance and competition as a motivator is linked to “fun” when respondents have indicated that “winning” is part of the fun for them.

Along similar lines, at the interpersonal level the main motivation to be with friends is linked to having fun by “playing with friends”. Being motivated for social reasons is linked with the fun element of “socialising”. At this level playing sport is fun when they can “play well as a team” and when “parents behave themselves”.

At the organisational level of the community sport club there are ways in which the (management of) the sport club can contribute to how playing sport can be fun. These ways were all related to coach behaviour and how coaches delivered the training and competition, including “getting game time”, “having well-organised practices” and “being friendly”. Overall, an inclusive and diverse club culture that includes volunteers, officials, parents, spectators, and players contributes significantly to higher levels of engagement and enjoyment in sport.

Also highlighted at the organisational level are main elements of how sport is delivered. Community club-based sport is largely based on a competition model delivered through volunteer run community clubs with traditional formats of training and intra-club competition formats with a focus on winning and talent development.

**Figure 2:**
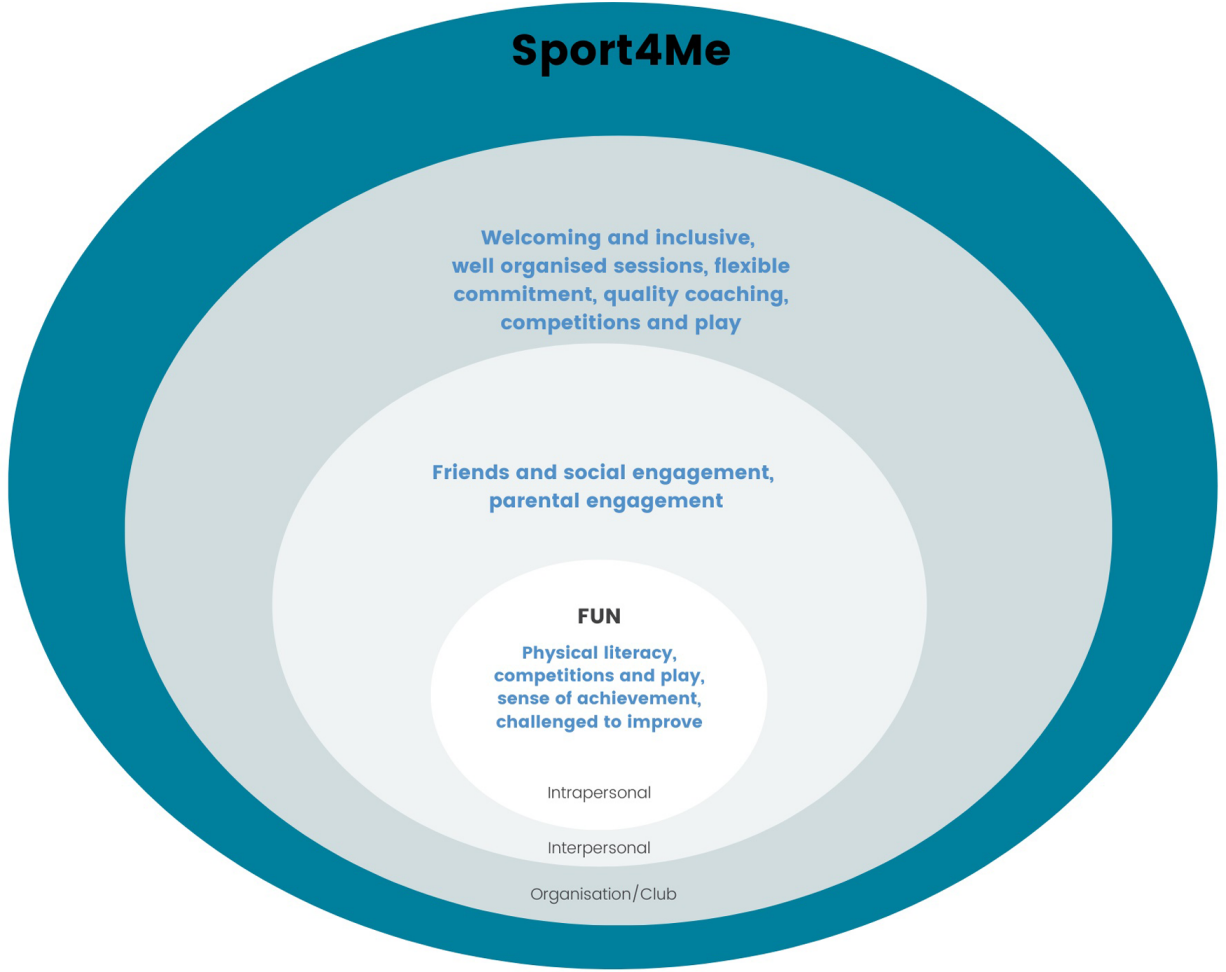
Sport4Me conceptual model.

From here we have developed a Sport4Me conceptual model (Figure 2) which depicts a sport model that has a person-centred approach. This model takes into account an understanding of the motivations and drivers to play sport and how the delivery of sport can further enhance participation, including retention and reengagement. The model utilises the domains of the socio-ecological model to highlight and summarise key aspects to be considered across the intrapersonal, interpersonal and organisational/club domains. At the core is a person-centred approach with “fun” as the key focus of the sport's design and delivery for participants.

## Conclusion

Participation in club-based sport remains largely based on the principles of competition and winning, ignoring wider societal changes that require a consumer driven approach to participation opportunities (10). As participation trends have resulted in uptake of different leisure-time activities, this has also impacted the number and therefore capacity of sports club volunteers.

It is clear that the traditional model of club-based sporting competitions does not cater well for societal changes that include the broader inclusion of individuals within the community and a wider range of preferences and motivations to play sport, that cannot be delivered in the traditional competitive club sport settings.

At the heart of our model sits “fun” and we have drawn on other research to further explain what “fun” means for participants of different age and gender. Overall, our main insight and as such, reconceptualization of how sport can be delivered, is to replace the focus of community sport structures on delivering competitions and “winners” of those competitions, to a wider perspective that positions the delivery of “fun sport” at the centre. This does not mean that club's or associations do away with competitions, but rather, from competitive (pathway driven) sport as the objective, to competitive sport as the principal means of delivering fun for all participants.

A shift from extrinsic motivations and drivers such as winning, to intrinsic factors of fun and enjoyment has the potential to assist with not only participation and retention (63, 64) but also has the potential to improve individual wellbeing (56).

In conclusion, the Sport4Me conceptual model has underlying principles that sports and/or community organisations can utilise in the development of participation, retention and re-engagement strategies and the implementation and delivery of a wider range of sport formats.

## References

[1] Eime R Charity M Westerbeek H. The Sport Participation Pathway Model (SPPM): a conceptual model for participation and retention in community sport. Int J Sport Policy Politics. (2022) 14(2):204–91. 10.1080/19406940.2022.2034913

[2] Eime R Charity M Harvey J Westerbeek H. Five-year changes in community-level sport participation, and the role of gender strategies. Front Sports Act Living. (2021) 3(281):710666. 10.3389/fspor.2021.710666 34712951 PMC8547161

[3] Eime R Harvey J Charity M. Sport participation settings: where and “how” do Australians play sport? BMC Public Health. (2020) 20(1):1344. 10.1186/s12889-020-09453-3 32883274 PMC7650524

[4] Eime R Harvey J Charity M Pankowiak A Westerbeek H. The impact of COVID-19 restrictions on Australians’ frequency and duration of participation in different types of sport and physical activity. BMC Sports Sci Med Rehabil. (2022) 14(1):42. 10.1186/s13102-022-00435-z 35313960 PMC8935269

[5] Westerbeek H Karg A. International sport business: current issues, future perspectives. New York: Routledge (2022).

[6] De Jonghe T. Sport en economie: samen in de spits, ed. r. Edition. Niewegein: Arko Sports Media (2019).

[7] Andrews D Silk M. Sport and neoliberalism: an effective-ideological articulation. J Pop Cult. (2018) 51(2):511–33. 10.1111/jpcu.12660

[8] Andrews D Ritzer G. The global in the sporting global. Glob Netw. (2007) 7(2):135–53. 10.1111/j.1471-0374.2007.00161.x

[9] Shilbury D Popi Sotiriadou K Christine Green B. Sport development. Systems, policies and pathways: an introduction to the special issue. Sport Manage Rev. (2008) 11(3):217–23. 10.1016/S1441-3523(08)70110-4

[10] Westerbeek H Eime R. The physical activity and sport participation framework—a policy model toward being physically active across the lifespan. Front Sports Act Living. (2021) 3:608593. 10.3389/fspor.2021.608593 34027402 PMC8138121

[11] Hertting K Karlefors I. We can’t get stuck in old ways”: Swedish sports club's integration efforts with children and youth in migration. Phys Cult Sport Stud Res. (2021) 92(1):32–42. 10.2478/pcssr-2021-0023

[12] English C. Toward sport reform: hegemonic masculinity and reconceptualizing competition. J Philos Sport. (2017) 44(2):183–98. 10.1080/00948705.2017.1300538

[13] Giulianotti R Coalter F Collison H Darnell SC. Rethinking Sportland: a new research agenda for the sport for development and peace sector. J Sport Soc Issues. (2019) 43(6):411–37. 10.1177/0193723519867590

[14] Jeanes R Spaaij R Penney D O’Connor J. Managing informal sport participation: tensions and opportunities. Int J Sport Policy Politic. (2019) 11(1):79–95. 10.1080/19406940.2018.1479285

[15] Rowe K Sherry E Osborne A. Recruiting and retaining girls in table tennis: participant and club perspectives. Sport Manage Rev. (2018) 21(5):504–18. 10.1016/j.smr.2017.11.003

[16] Eime R Harvey J Charity M Westerbeek H. Participation of Australian women and girls in traditionally male-dominated sports 2016–2018. Int J Sport Policy Politic. (2022) 14(3):545–61. 10.1080/19406940.2022.2090995

[17] Pill S Doolittle S Baldock R. Chapter 1: TGfu: a model for the teaching of games with a changed focus in games teaching- a commentary. In: Pill S Griffin L Gambles E-AF, editor. Perspectives on teaching games for understanding. Routledge (2023).

[18] Richards R. Aussie Sports. (2020). p. 1–9. (cited Oct 29, 2022). Available from: https://www.clearinghouseforsport.gov.au/kb/aussie-sports

[19] Richards R May C. Modified Sports. (2022). Available from: https://www.clearinghouseforsport.gov.au/kb/modified-sports

[20] Light R. Game sense: pedagogy for performance, participation and enjoyment. London, UK: Routledge (2012).

[21] Pill S SueSee B Rankin J Hewitt M. The spectrum of coaching styles. London, UK: Routledge (2022).

[22] Australian Sports Commission. Game sense: perceptions and actions research report. Canberra: Australian Sports Commission (1996).

[23] Australian Sports Commission. Game sense (video). Canberra: Australian Sports Commission (1997).

[24] de Duyn N. Game sense: Developing thinking players. A presenters guide and workbook. Canberra: Australian Sports Commission (1997).

[25] Barrett B Den Duyn N Durham D Goodman S MacGraw D Murphy G. Game sense cards: 30 games to develop thinking players. Canberra: Australian Sports Commission (1999).

[26] Stone J Rothwell M Shuttleworth R Davids K. Exploring sports coaches’ experiences of using a contemporary pedagogical approach to coaching: an international perspective. Qual Res Sport Exerc Health. (2021) 13(4):639–57. 10.1080/2159676X.2020.1765194

[27] Agnew D Pill S Drummond M. Investigating the elements that encourage or inhibit the participation of children and youth in Australian Football. Ann Leis Res. (2016) 19(1):27–46. 10.1080/11745398.2015.1036898

[28] Agnew D Pill S. The role of the coach in player retention and attrition. In: Toms M Jeanes R, editors. Routledge handbook of coaching children in sport. Routledge (2022).

[29] McLeroy K Bibeau D Steckler A Glanz K. An ecological perspective on health promotion programs. Health Educ Q. (1988) 15(4):351–77. 10.1177/109019818801500401 3068205

[30] Biernat E Skrok Ł Majcherek D Nałęcz H. Socioecological profile of active adults. Sport as a whole-life choice. Phys Cult Sport Stud Res. (2020) 85(1):59–76. 10.2478/pcssr-2020-0007

[31] Eime RM Casey MM Harvey JT Sawyer NA Symons CM Payne WR. Socioecological factors potentially associated with participation in physical activity and sport: a longitudinal study of adolescent girls. J Sci Med Sport. (2015) 18:684–90. 10.1016/j.jsams.2014.09.012 25308630

[32] Devís-Devís J Beltrán-Carrillo VJ Peiró-Velert C. Exploring socio-ecological factors influencing active and inactive Spanish students in years 12 and 13. Sport Educ Soc. (2015) 20(3):361–80. 10.1080/13573322.2012.754753

[33] Fowlie J Eime RM Griffiths K. Barriers to adolescent female participation in cricket. Ann Leis Res. (2021) 24(4):513–31. 10.1080/11745398.2019.1710716

[34] Lewko J Greendorfer S. Family influences in sport specialization of children and adolescents. In: Smoll F Magill R Ash M, editors. Children in sport. Champaign, Illinois: Human Kinetics (1988). p. 288–300.

[35] MacDougall C Shilller W Darbyshire P. We have to live in this future. Early Child Dev Care. (2004) 17(4):369–87. 10.1080/0300443032000153426

[36] MacDougall C Schiller W Darbyshire P. Through children’s eyes: The good, the bad and the ugly about play and sport, in Be Active ‘07. (2007).

[37] Gallant F O'Loughlin J Brunet J Sabiston C Belanger M. Childhood sports participation and adolescent sport profile. Pediatrics. (2017) 140(6):e20171449. 10.1542/peds.2017-1449 29133575

[38] Atkins M Johnson D Force E Petrie T. Peers, parents, and coaches, oh my! THe relation of hte motivational climate to boys’ intention to continue in sport. Psychol Sport Exerc. (2015) 16(Part 3):170–80. 10.1016/j.psychsport.2014.10.008

[39] Pill S Harvey S. A narrative review of children’s movement competency research 1997-2017. Phys Cult Sport Stud Res. (2019) 81(1):47–74. 10.2478/pcssr-2019-0005

[40] Light RL Harvey S Memmert D. Why children join and stay in sports clubs: case studies in Australian, French and German swimming clubs. Sport Educ Soc. (2013) 18(4):550–66. 10.1080/13573322.2011.594431

[41] Eime R Harvey J Charity M Pankowiak A Westerbeek H. The changing context of participation in organised club-based sport and the motivations to play. J Glob Sport Manag. (2022), Submitted.

[42] Lim SY Warner S Dixon M Berg B Kim C Newhouse-Bailey M. Sport participation across national contexts: a multilevel investigation of individual and systemic influences on adult sport participation. Eur Sport Manag Q. (2011) 11(3):197–224. 10.1080/16184742.2011.579993

[43] Stenner BJ Buckley JD Mosewich AD. Reasons why older adults play sport: a systematic review. J Sport Health Sci. (2020) 9(6):530–41. 10.1016/j.jshs.2019.11.003 33308804 PMC7749227

[44] Jenkin CR Eime RM van Uffelen JGZ Westerbeek H. How to re-engage older adults in community sport? Reasons for drop-out and re-engagement. Leis Stud. (2021) 40(4):441–53. 10.1080/02614367.2021.1888310

[45] Deelen I Ettema D Kamphuis CBM. Sports participation in sport clubs, gyms or public spaces: how users of different sports settings differ in their motivations, goals, and sports frequency. PLoS One. (2018) 13(10):e0205198. 10.1371/journal.pone.0205198 30296286 PMC6175514

[46] Eime R Harvey J Charity M Weterbeek H. What is fun about playing sport? Submitted to Annals of Leisure Research. (2022).

[47] Eime RM Harvey JT Sawyer NA Craike MJ Symons CM Polman RC Understanding the contexts of adolescent female participation in sport and physical activity. Res Q Exerc Sport. (2013) 84(2):157–66. 10.1080/02701367.2013.784846 23930541

[48] Visek A Achrati S Mannix H McDonnell K Harris B DiPietro L. The fun integration theory: toward sustaining children and adolescent sport participation. J Phys Act Health. (2015) 12(3):424–33. 10.1123/jpah.2013-0180 24770788 PMC4201634

[49] Collins K Barcelona R. Keep ‘em playing: strategies for building positive sport experiences. Strategies. (2018) 31(5):8–14. 10.1080/08924562.2018.1490231

[50] Foley BC Rose C Owen KB Reece LJ. Linking sports registration information and player feedback to enhance netball participation. BMC Sports Sci Med Rehabil. (2021) 13(1):59. 10.1186/s13102-021-00286-0 34103084 PMC8188710

[51] Dixon N. The proper place for external motivations for sport and why they need not subvert its internal goods. Sport Ethics Philos. (2018) 12(4):361–74. 10.1080/17511321.2018.1498908

[52] Egli T Bland HW Melton BF Czech DR. Influence of age, sex, and race on college students’ exercise motivation of physical activity. J Am Coll Health. (2011) 59(5):399–406. 10.1080/07448481.2010.513074 21500059

[53] Soares J Antunnes H van den Tillaar R. A comparison between boys and girls about motivations for the participation in school sport. J Phys Educ Sport. (2013) 13(3):303–7. 10.7752/jpes.2013.03050

[54] Moradi J Bahrami A Amir D. Motivation for participation in sports based on athletes in team and individual sports. Phys Cult Sport. (2020) 85(1):14–21. 10.2478/pcssr-2020-0002

[55] Jakobsen A Evien E. Gender differences in motives for particiation in sports and exercise among Norwegian adolescents. Balt J Sport Health Sci. (2018) 10(2):92–101. 10.29359/BJHPA.10.2.10

[56] Jetzke M Mutz M. Sport for pleasure, fitness, medals or slenderness? Differential effects of sports activities on well-being. Appl Res Qual Life. (2020) 15(5):1519–34. 10.1007/s11482-019-09753-w

[57] Murray R Koulanova A Sabiston C. Understanding girls’ motivation to participate in sport: the effects of social identity and physical self-concept. Front Sports Act Living. (2021) 3:787334. 10.3389/fspor.2021.787334 35088047 PMC8787279

[58] Eime R Harvey J. Sport participation across the lifespan: Australian trends and policy implications. In: Dionigi R Gard M, editors. Sport and physical activity across the lifespan. London, UK: Palgrave Macmillan (2018). p. 23–43.

[59] Kahneman D. The riddle of experience versus memory, T. conference, Editor. (2010).

[60] Sharot T. The influential mind: What the brain reveals about our power to change others. New York: Henry Holt and Company (2017).

[61] Bauman A Owen N Rushworth R. Recent trends and socio-demographic determinants of exercise participation in Australia. Community Health Stud. (1990) 14(1):19–26. 10.1111/j.1753-6405.1990.tb00016.x 2331859

[62] Eime RM Casey MM Harvey JT Sawyer NA Symons CM Payne WR. Socioecological factors potentially associated with participation in physical activity and sport: a longitudinal study of adolescent girls. J Sci Med Sport. (2015) 18(6):684–90. 10.1016/j.jsams.2014.09.012 25308630

[63] Eime R Harvey J Charity M Westerbeek H. Longitudinal trends in sport participation and retention of women and girls. Front Sports Act Living. (2020) 2:39. 10.3389/fspor.2020.00039 33345031 PMC7739630

[64] Eime R Casey M Harvey J. Developing sport for girls and adolescents. In: Sherry E Rowe K, editors. Developing sport for women and girls. London: Routledge (2020). p. 19–32.

